# Toll-like receptor 3-mediated inflammation by p38 is enhanced by endothelial nitric oxide synthase knockdown

**DOI:** 10.1186/s12964-019-0345-3

**Published:** 2019-04-15

**Authors:** Stephen R. Koch, Hyehun Choi, Eric H. Mace, Ryan J. Stark

**Affiliations:** 1 Department of Pediatrics, Vanderbilt University Medical Center, 2200 Children’s Way, 5121 Doctors’ Office Tower, Nashville, TN 37232-9075 USA; 2 Department of Surgery, Vanderbilt University Medical Center, Nashville, TN 37232 USA

**Keywords:** eNOS, TLR3, Inflammation, Endothelial dysfunction, p38

## Abstract

**Background:**

Vascular dysfunction is commonly seen during severe viral infections. Endothelial nitric oxide synthase (eNOS), has been postulated to play an important role in regulating vascular homeostasis as well as propagation of the inflammatory reaction. We hypothesized that the loss of eNOS would negatively impact toll-like receptor 3 (TLR3) signaling and worsen vascular function to viral challenge.

**Methods:**

Human microvascular endothelial cells (HMVECs) were exposed to either control or eNOS siRNA and then treated with Poly I:C, a TLR3 agonist and mimicker of dsRNA viruses. Cells were assessed for protein-protein associations, cytokine and chemokine analysis as well as transendothelial electrical resistance (TEER) as a surrogate of permeability.

**Results:**

HMVECs that had reduced eNOS expression had a significantly elevated increase in IL-6, IL-8 and IP-10 production after Poly I:C. In addition, the knockdown of eNOS enhanced the change in TEER after Poly I:C stimulation. Western blot analysis showed enhanced phosphorylation of p38 in sieNOS treated cells with Poly I:C compared to siControl cells. Proximity ligation assays further demonstrated direct eNOS-p38 protein-protein interactions. The addition of the p38 inhibitor, SB203580, in eNOS knockdown cells reduced both cytokine production after Poly I:C, and as well as mitigated the reduction in TEER, suggesting a direct link between eNOS and p38 in TLR3 signaling.

**Conclusions:**

These results suggest that reduction of eNOS increases TLR3-mediated inflammation in human endothelial cells in a p38-dependent manner. This finding has important implications for understanding the pathogenesis of severe viral infections and the associated vascular dysfunction.

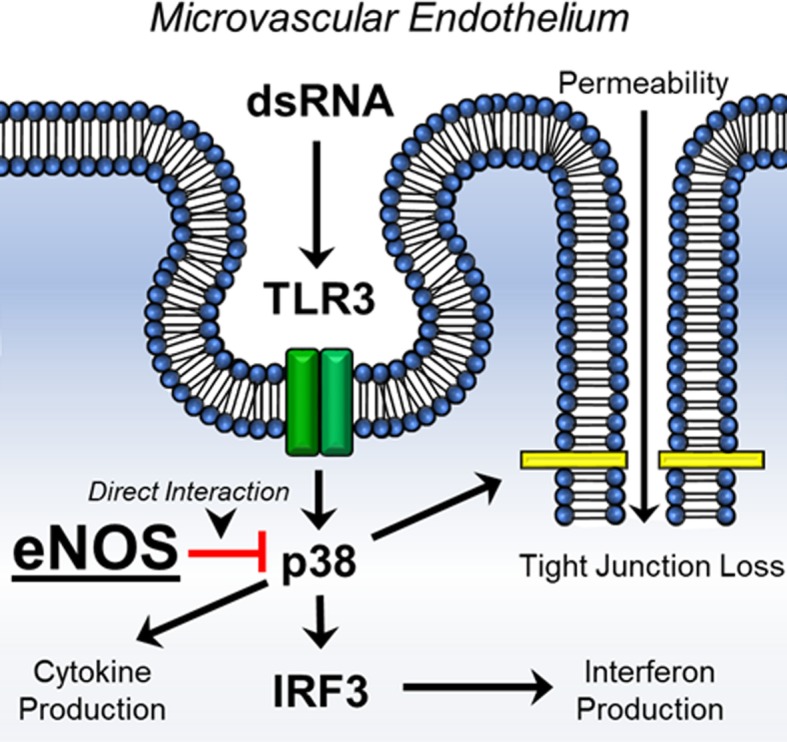

**Electronic supplementary material:**

The online version of this article (10.1186/s12964-019-0345-3) contains supplementary material, which is available to authorized users.

## Background

The endothelium is a layer of cells that cover the entire vasculature and is responsible for maintaining vascular homeostasis [1]. It serves as an interface between the blood and the tissue. However, when the layer of endothelial cells is disturbed, gaps form within the monolayer allowing for passage of fluids into the tissue and disrupting the protective integrity; this phenomenon is known as barrier dysfunction [2]. While many endogenous and exogenous triggers can induce barrier dysfunction, several key receptors on the surface of the endothelium are responsible for cellular activation in the setting of systemic pathogen challenge. These receptors are known as toll-like receptors (TLRs) [3]. Activation of toll-like receptors by pathogens induces significant injury, setting off a cascade of inflammation within the endothelium and surrounding environment. This inflammation results in the production of pro-inflammatory cytokines and chemokines, changes within vascular tone, leukocyte adhesion and capillary leak [4]. In the clinical setting, the constellation of these findings is known as sepsis, which is an exaggerated response to an infectious challenge.

Derangements in the endothelium related to an infectious stimulus have often portrayed nitric oxide (NO) as the mediator of the resulting pathology [5]. Within the endothelium, NO is produced mainly by the constitutively expressed enzyme, endothelial nitric oxide synthase (eNOS) [6]. Both the over and under production of NO during inflammatory challenge has been linked to alterations in endothelial barrier dysfunction, though its role remains controversial, especially in the setting of sepsis and severe, acute infections [7]. And while there certainly is a role for NO in that pathology, it has been shown that eNOS can also alter inflammatory signaling in response to infectious challenge in an NO-independent manner [8]. In this setting, the modulatory effects of eNOS on inflammation appear to be related through regulation of the p38 mitogen activated protein kinase (MAPK) pathway in TLR4 activation. Though homology exists among all TLRs, within endothelial cells, TLR4 activation is dependent more on cell surface activation while TLR3, the TLR responsible for activation by double stranded RNA (dsRNA) viruses, requires endocytosis prior to inflammatory stimulation.

While activation of MAPKs, specifically p38, are a well-known phenomenon after TLR4 stimulation, it is less clear of the role they play in TLR3 signaling [9]. Specifically, a primary endpoint of TLR3 activation is the production of interferon-beta, which is necessary to combat viral infections. It has been suggested that p38 can help regulate interferon signaling by acting as a courier for interferon regulatory factor (IRF) into the nucleus for transcription [10]. Given the previously described relationship between eNOS and p38, as well as a postulated relationship between IRF and p38, we hypothesized that eNOS negatively regulates p38 activity and as such, loss of eNOS would potentiate inflammation mediated by Poly I:C, a TLR3 agonist, in a p38-dependent manner. We further hypothesized that this dysregulated p38 activation would exacerbate IRF signaling and in total, contribute to the global endothelial inflammation seen during severe viral infections. These finding would provide new understandings into the protective role of eNOS in endothelial inflammation as well as provide mechanistic insights into the development of endothelial dysfunction in viral sepsis.

## Methods

### Cells and culture

Pooled, neonatal dermal human microvascular endothelial cells (HMVECs) were purchased from Lonza (Basel, Switzerland). HMVECs were grown in Endothelial Growth Media-2 (Lonza) supplemented with 5% fetal bovine serum (FBS). Cells were plated at a density of approximately 30,000 cells/cm^2^ and grown to confluence. For a positive control for iNOS expression, murine vascular smooth muscle cells from strain C57BL/6 were grown in Dulbecco’s Modified Eagle’s Medium (DMEM) supplemented with 10% FBS. Experiments were conducted between the 2nd and 5th passages. Media was exchanged every 3 days.

### Agonist and inhibitor reagents

The following reagents and concentrations were used in experiments: 10 μg/mL Poly I:C (Sigma Aldrich, St. Louis, MO, USA), 100 μmol/L L-NG-Nitroarginine methyl ester (L-NAME, Sigma Aldrich, St. Louis, MO, USA), 10 μmol/L SB203580 (Cell Signaling Technology, Danvers, MA, USA). For a positive control of iNOS induction, 10 ng/mL tumor necrosis factor alpha (TNFα, Sigma Aldrich, St. Louis, MO, USA) was used. Agonists, inhibitors and vehicle controls were provided to cells in the presence of culture media.

### Western blot and electrophoresis

For siRNA experiments, cell lysates were collected at 90 min after agonist exposure. This time point was based on previously published data of protein phosphorylation kinetics [11]. In a separate set of experiments examining for NOS proteins, cell lysates were collected at 90 min, 6 h and 16 h after agonist exposure. In addition, for a positive control for iNOS expression, murine vascular smooth muscle cells were exposed to TNFα or vehicle control for 24 h prior to lysate collection. Protein extracts (50 μg/sample) were separated by SDS electrophoresis on a polyacrylamide gel (10%) and transferred to nitrocellulose membranes. Membranes were blocked with Odyssey Blocking Buffer (LI-COR Biosciences, Lincoln, NE, USA) for 1 h at room-temperature. Membranes were incubated with primary antibodies overnight at 4 °C on a rocker. Antibodies were as follows: IKKαβ, phospho-IKKβ, p-p38, p38, p-ERK, ERK, p-IRF7, IRF7, p-eNOS (S1177), eNOS (Cell Signaling Technology, Danvers, MA, USA), iNOS (R&D Systems, Minneapolis, MN, USA), IRF3 (Santa Cruz Biotechnology, Inc., Dallas, TX, USA), p-IRF3 (EMD Milipore, Billerica, MA, USA), and αTubulin (Vanderbilt Antibody Core, Nashville, TN, USA). Afterwards, membranes were incubated with fluorescent secondary antibodies and analyzed using the Odyssey Imaging System (LI-COR Biosciences, Lincoln, NE, USA). Protein quantification was performed via densitometry and normalized as a ratio of expressed protein to αTubulin or phosphorylated protein to respective total protein.

### siRNA transfection

HMVECs were treated with siRNA (scrambled siControl, sieNOS) according to the manufacturer’s recommendations. In brief, siRNA was procured from Dharmacon (Lafayette, CO, USA). siRNA (25 nmol/L) was incubated with Dharmafect (Dharmacon) in serum-free medium for 20 min. The resultant complex of siRNA-Dharmafect was added to the cells in 5% FBS media without antibiotics for 6 h. Afterwards, the transfection media was replaced with complete media including antibiotics for another 66 h for a total of 72 h siRNA incubation time prior to agonist exposure. For conditions using TEER, cells were exposed to siRNA for 6 h with removal of siRNA containing media afterwards and replacement of fresh media. Cells were permitted to remain undisturbed for another 42 h. Then cells were plated on the CellZScope and allowed to settle for 24 h to achieve confluence and complete 72 h siRNA incubation time prior to agonist exposure. Percent protein knockdown was determined by western blot analysis as described above.

### Cytokine and chemokine production

Supernatants from cells cultures were collected at the completion of the agonist exposure (6 or 16 h) based on the timing of cytokine secretion from previously published data [12]. Collected supernatants were stored at − 80 °C until analyzed. Supernatant IL-6 (eBioScience, San Diego, CA, USA), IL-8, IP-10 and soluble VCAM (sVCAM) (R&D Systems, Minneapolis, MN, USA) concentrations were assessed using a commercially available enzyme-linked immunosorbent assay (ELISA) kit according to the manufacturer’s specifications.

### Transendothelial electrical resistance (TEER) assay

Determination of TEER was performed utilizing the CellZScope2 (nanoAnalytics GmbH, Münster, Germany). For assays involving siRNA, treatment of siRNA was done prior to plating cells to reduce the impact of washing on monolayer formation and resistance. HMVECs were plated on ThinWell Cell Culture inserts (0.4 μm pore diameter, Greiner Bio-One, Monroe, NC) coated with poly-L-lysine (2x) for 20 min then washed with Dulbecco’s phosphate-buffered saline (D-PBS), glutaraldehyde 50% (1000x) for 15 min then washed with D-PBS, and lastly gelatin (40x) then washed again with D-BPS. Afterwards 70% ethanol was applied for 30 min and washed followed by an aldehyde scavenger for another 30 min in medium and then removed. Cells were then plated at 40,000 cells/well and allowed to grow to confluence over 24 h. Afterwards, cells were treated with Poly I:C for 16 to 24 h and examined for TEER in 1 h increments. In some experiments, 30 min prior to Poly I:C exposure, cells were treated with L-NAME, SB203580 or vehicle control (DMSO < 1%) and remained for the duration of the Poly I:C exposure.

### Proximity ligation assay (PLA)

Proximity Ligation Assays were performed using a commercially available kit according to the manufacturer’s instructions (Duolink PLA, Sigma Aldrich, St. Louis, MO, USA). In brief, 96-well glass bottom plates were utilized for the experiment and pretreated with poly-L-lysine, glutaraldehyde 50%, gelatin and an aldehyde scavenger in the same concentrations and times and described above. HMVECs were then plated and exposed to siRNA for 72 h total incubation time as previously described. Cells were then exposed to Poly I:C for 90 min. Afterwards, the media containing Poly I:C was removed, cells were washed with PBS and exposed to a formaldehyde containing fixing solution (8 parts water, 1 part 40% formaldehyde, 1 part 10x PBS) for 15 min and permeabilized with 0.1% Triton X-100 in PBS for 15 min. The solution was then removed and 40 μL of Duolink Blocking Solution (Sigma Aldrich, St. Louis, MO, USA) was placed in each well and incubated at 37 °C for 1 h. The blocking solution was then replaced with Duolink Antibody Diluent containing the following primary antibodies at a 1:100 dilution: eNOS rabbit mAb (clone #D9A5L, Cell Signaling Technology, Danvers, MA, USA), p38α mouse mAb (clone #9F12, Novus Biologicals, Littleton, CO, USA). Plates were then sealed and incubated overnight at 4 °C.

The next day, the primary antibodies were removed, and the cells were washed with Duolink Wash Buffer. Duolink PLA probes (anti-rabbit secondary antibody with plus oligonucleotides and anti-mouse secondary antibody with minus oligonucleotides) were diluted in a 1:5 ratio in Duolink Antibody Diluent and 40 μL of probe containing solution was added to each well for 1 h at 37 °C. Kit ligase was added at a 1:40 dilution to Duolink Ligation Buffer, which contained pre-mixed concentrations of bridging oligonucleotides, and added to each well for 30 min at 37 °C. Next, cells were washed and kit provided polymerase was added in a 1:80 dilution in Duolink Amplification Buffer and applied to each well for 100 min at 37 °C. The amplification buffer included pre-mixed concentrations of Duolink Orange fluorescent probes (ex 554/em 576). Cells were then washed and Duolink Wash Buffer containing 1 drop NucBlue (ex 360/em 460, Thermo Fischer Scientific, Waltham, MA, USA) per mL of buffer was added to each well at room temperature for 10 min. The wash buffer was then removed and replaced with 120 μL of Live Cell Imaging Solution (Thermo Fischer Scientific, Waltham, MA, USA) and imaged at the appropriate wavelength using an inverted confocal microscope (Zeiss LSM 880, Carl Zeiss AG, Oberkochen, Germany). Fluorescent reactions and cell number were counted by averaging two random fields per well using ImageJ software (National Institutes of Health, Bethesda, MD, USA).

### Intracellular nitric oxide quantification

NO was determined using the fluorescent probe 4,5-Diaminofluorescein Diacetate (DAF-2 DA, Sigma Aldrich, St. Louis, MO, USA). Cells were grown on 96-well glass bottom plates and treatments applied as described above. After reaching confluence, cells were exposed to Poly I:C for 90 min or 6 h. Cells treated with no agonist or L-NAME for 90 min served as controls. Afterwards, supernatants were removed, cells were washed with media and exposed to 5 μmol/L DAF-2 DA for 40 min in the dark at 37 °C. The media containing DAF-2 DA was then removed and replaced with fresh phenol-red free media and the cells remained in the dark for an additional 30 min at 37 °*C.* Media was then replaced with 120 μL of Live Cell Imaging Solution (Thermo Fischer Scientific, Waltham, MA, USA) supplemented with 5% FBS and 5 mmol of glucose and imaged for retained intracellular probe (ex 485/em 520) using an inverted confocal microscope (Zeiss LSM 880, Carl Zeiss AG, Oberkochen, Germany). Treated wells (L-NAME or agonist) were normalized against fluorescent intensity in the control, untreated wells.

### Gene profiles of human biopsies

Gene array profiles of omental adipose tissue biopsies were obtained from the publicly available National Centers for Biotechnology Information (NCBI) Gene Expression Omnibus (GEO) database. Values were obtained from GEO Dataset (GSE20950) initially collected by Hardy OT, et al. “Morbidly obese insulin-resistant patients: omental and subcutaneous adipose tissue” [13]. The database was queried for NOS3 (eNOS, ID: NM_000603), MAPK14 (p38, ID: L35253) and TLR3 (TLR3, ID: NM_003265) from samples run on Affymetrix Human Genome U133 Plus 2.0 Array.

### Statistical analysis

For endothelial cell culture experiments, data are expressed as means ± SE of multiple, individual experiments. Comparisons of treatment groups and conditions were done via unpaired t-test for single comparisons and one-way ANOVA, with Bonferroni correction, for multiple-group comparisons. For gene profile expression of biopsy samples, data are expressed as medians (5–95%) ± SE of individual cohorts. Comparisons of groups were done via a Mann-Whitney test and linear regression for direct expression comparison with 95% CI. All analysis was done using GraphPad Prism 5.03 statistical software (GraphPad Software Inc., La Jolla, CA). A *p* value of < 0.05 was considered statistically significant.

## Results

### Knockdown of eNOS potentiates cytokine and chemokine release after TLR3 activation

**Fig. 1:**
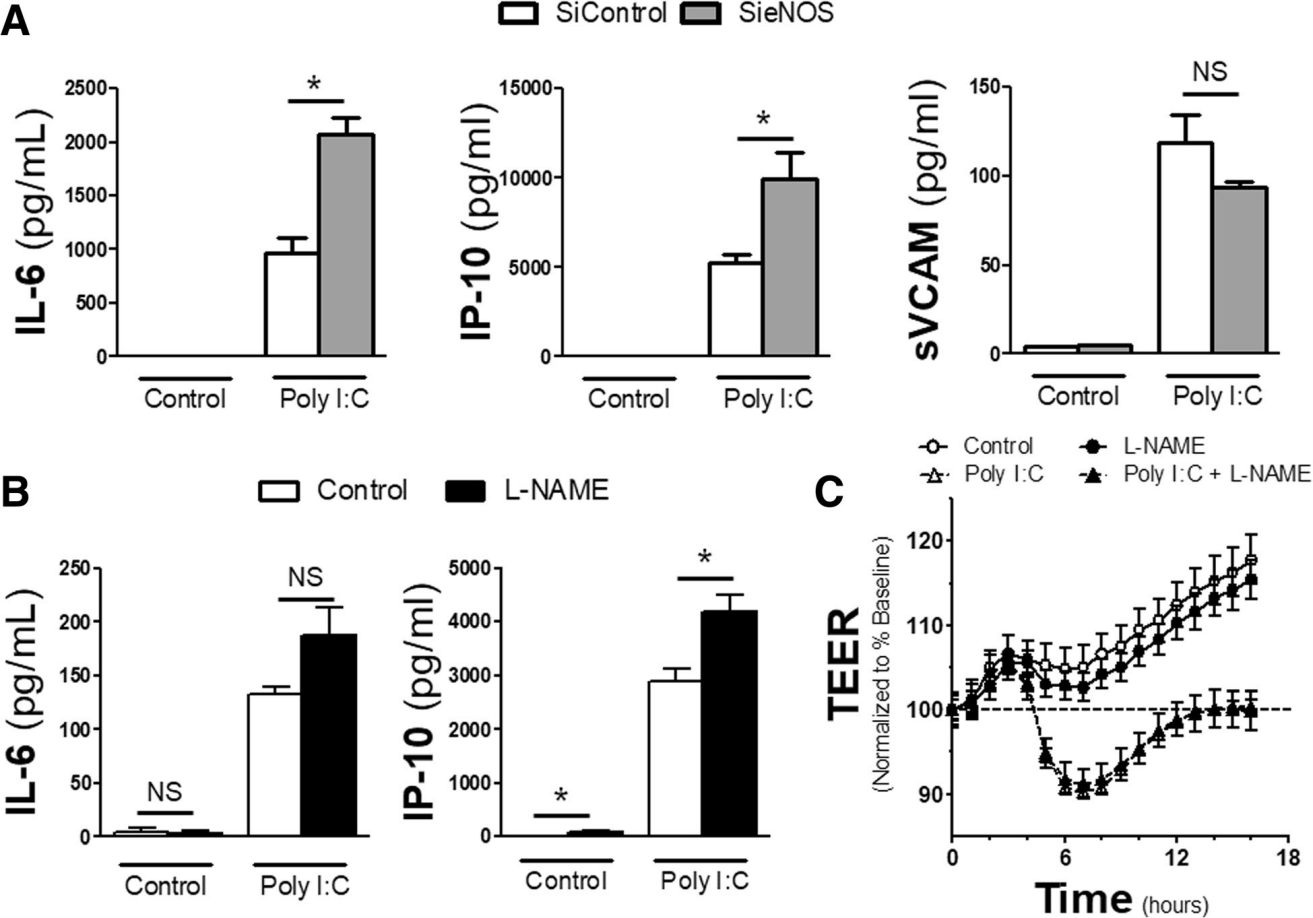
*Effect of eNOS siRNA and L-NAME on cytokines*: **a** HMVECs were treated with 72 h of siRNA scrambled (siControl) or eNOS (sieNOS) and then treated with Poly I:C (TLR3 agonist, 10 μg/mL) for 16 h. Supernatants were collected and the amount of IL-6, IP-10 and sVCAM produced among treatment groups was measured by ELISA (*n* = 4 per group). **b** HMVECs were exposed to L-NAME (100 μmol) or vehicle control (DMSO < 0.1%) 30 min prior to Poly I:C (10 μg/mL) treatment. Supernatants were then collected after 16 h of Poly I:C treatment and measured for IL-6 and IP-10 by ELISA (n = 4 replicates per group). **c** HMVECs were plated on transwells for 24 h. Baseline levels for normalization are shown at time 0. Cells were pretreated with L-NAME for 30 min and then Poly I:C afterwards at the doses listed in B. Change in trans-endothelial electrical resistance (TEER) from baseline (---) was determined as hourly measurements over 16 h from Poly I:C exposure (n = 4 replicates per group). * = *p* < 0.05 between compared groups, NS = non-significant

Data has demonstrated that knockdown of eNOS potentiates TLR4-mediated inflammation [8]. However, in endothelial cells, TLR4 activation relies primarily on the myeloid differentiation primary response 88 (MyD88) pathway for inflammatory signal transduction, which occurs on the cell surface. This is in comparison to the TIR-domain-containing adapter-inducing interferon-β (TRIF) signaling pathway utilized by endosomal TLRs, such as TLR3. To examine if eNOS served a protective function in other TLR-related mechanisms, we used siRNA to reduce the amount of endogenous eNOS within human microvascular endothelial cells (HMVECs). After 72 h of total siRNA exposure, cells were challenged with Poly I:C, a TLR 3 agonist, or media control for 16 h and supernatants were collected. Reduction of eNOS produced a near doubling of both IL-6 and IP-10 production after Poly I:C exposure compared to cells treated with scrambled siRNA (siControl) (Fig. 1a). However, the inflammatory potentiation by the loss of eNOS was not uniform, as the sieNOS group had the same production of soluble VCAM compared to the siControl group after Poly I:C, suggesting differential regulation of inflammation by eNOS. To examine how much of this effect was nitric oxide (NO) mediated, in a separate set of experiments, cells were pretreated with the global NOS inhibitor, L-NAME, 30 min prior to and for the duration of Poly I:C treatment. Under these conditions, the application of L-NAME led to some enhanced IP-10 and IL-6 production in Poly I:C exposed cells compared to vehicle controls, though it only reached statistical significance for IP-10 levels (Fig. 1b). In addition, the application of L-NAME had no effect on Poly I:C induced changes in the trans-endothelial electrical resistance (TEER) of the monolayer of cells (Fig. 1c).

### Reduction of eNOS potentiates loss in endothelial barrier integrity

**Fig. 2:**
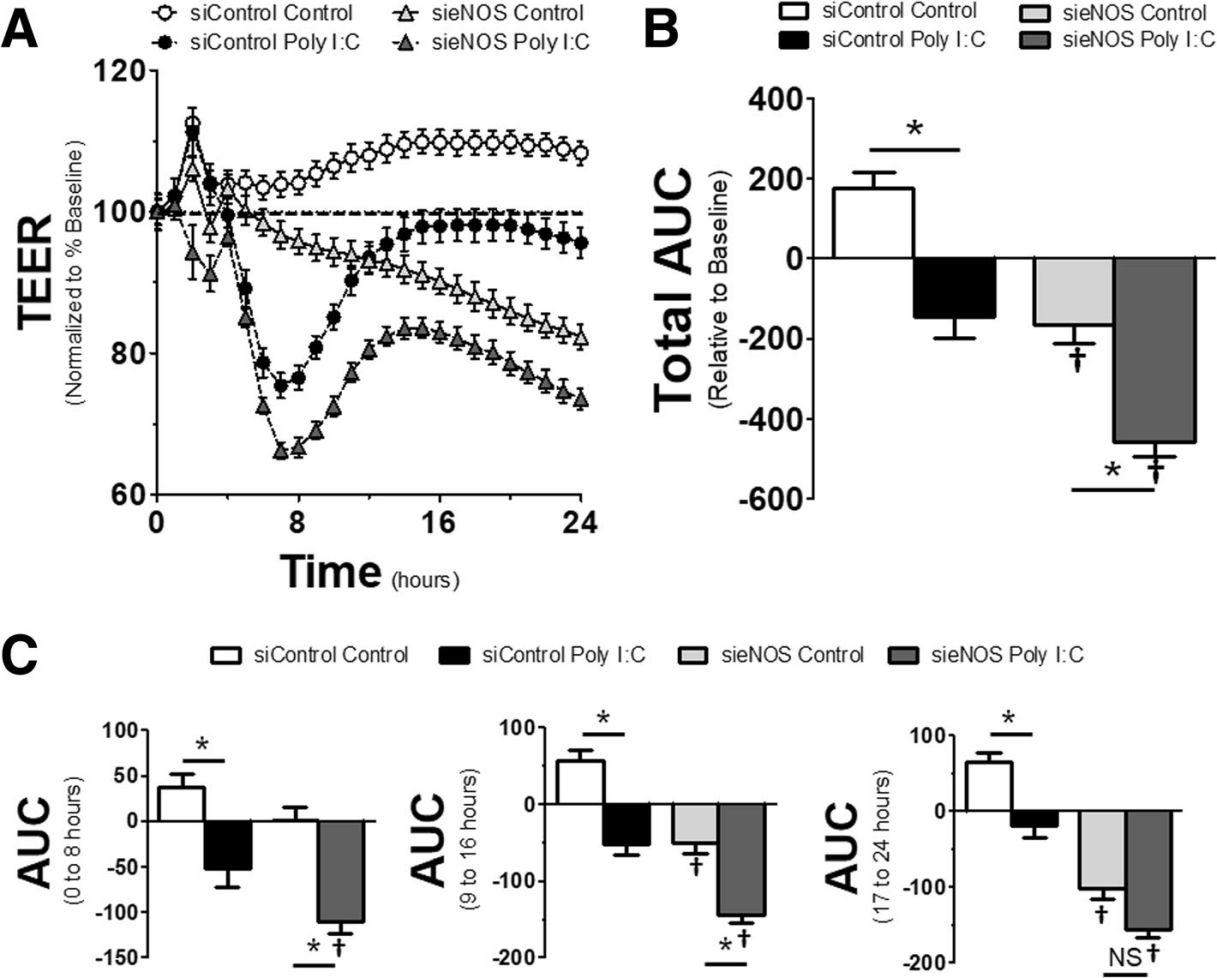
*eNOS knockdown induced changes in endothelial monolayer resistance*: **a** HMVECs were exposed to siRNA for 72 h (siControl or sieNOS) and then treated with Poly I:C (10 μg/mL). Trans-endothelial electrical resistance (TEER) was determined by measuring resistance across the monolayer at 1 h intervals for a total of 24 h across the respective groups and normalized to the resistance 1 h prior to agonist treatment. **b** Total area under the curve (AUC) for changes in resistance. AUC was determined by aggregate change in resistance (increase = positive, decrease = negative) relative to baseline for all data points collected. **c** AUC for specific time intervals of 0 to 8 h (*left)*, 9 to 16 h (*center*) and 17 to 24 h (*right*) (*n* = 4 replicates per group). * = *p* < 0.05 between compared groups, † = *p* < 0.05 between group compared to respective siControl group, NS = non-significant

It has been suggested that both TLR4 activation and eNOS can induce changes in endothelial barrier integrity, leading to enhanced permeability across the monolayer. While the postulated mechanisms responsible for TLR4-mediated barrier dysfunction are numerous, the role of eNOS in maintaining barrier function is less clear. This is especially true considering the data above showing no effect of L-NAME, yet other data showing that NO also has the ability to cause barrier dysfunction [14]. To test the role of eNOS in TLR3-induced barrier dysfunction, HMVECs were exposed to siRNA to eNOS or scrambled controls for 72 h. Then cells were challenged with Poly I:C and monitored for dynamic alterations in barrier integrity by examining the change in TEER across the monolayer. Figure 2a shows the dynamic output of these treatments. Stimulation of endothelial cells by Poly I:C induced a slow, but steady reduction in TEER, indicating a loss of endothelial integrity and increased permeability, that reached its maximum potential around 7 h after initial stimulation. Afterwards, there was some recovery in endothelial integrity at a similar rate, achieving near baseline conditions at approximately 16 h. However, in HMVECs with knockdown of eNOS, endothelial barrier integrity was reduced once the cells reached confluence and continued to steadily decline over the duration of the experiment despite not being exposed to any TLR3 agonist. In sieNOS cells treated with Poly I:C, the cells had a greater loss of resistance after Poly I:C challenge and had an impaired recovery. These dynamic effects are shown overall via an area under the curve (AUC) (Fig. 2b). The AUC was calculated by taking the percent change in resistance (increase or positive and decrease or negative) relative to the baseline for each TEER measurement (see dashed line in Fig. 2a) and summing them together to get the area of percent change over time.

The AUC was further broken down into time intervals and showed dynamic changes in endothelial integrity over time. In this data, shown in Fig. 2c, while Poly I:C induced significant changes in endothelial barrier integrity in the first 8 h after TLR3 agonist exposure, the changes between the siControl and sieNOS groups for control conditions were not statistically significant. However, in the 9 to 16 h interval, while the effect of Poly I:C still induced a significant loss in endothelial resistance, the sieNOS group had reduced resistance compared to the siControl group in both control and TLR3 stimulated conditions. This difference increased in the 17 to 24 h time frame where the effect of Poly I:C was still statistically significant in the siControl group, yet not different in the sieNOS group due to the persistent loss of endothelial integrity in HMVECs with eNOS knockdown, even in the absence of TLR3 stimulation.

### p38 and interferon regulatory factor are enhanced by eNOS knockdown and eNOS directly interacts with p38

**Fig. 3:**
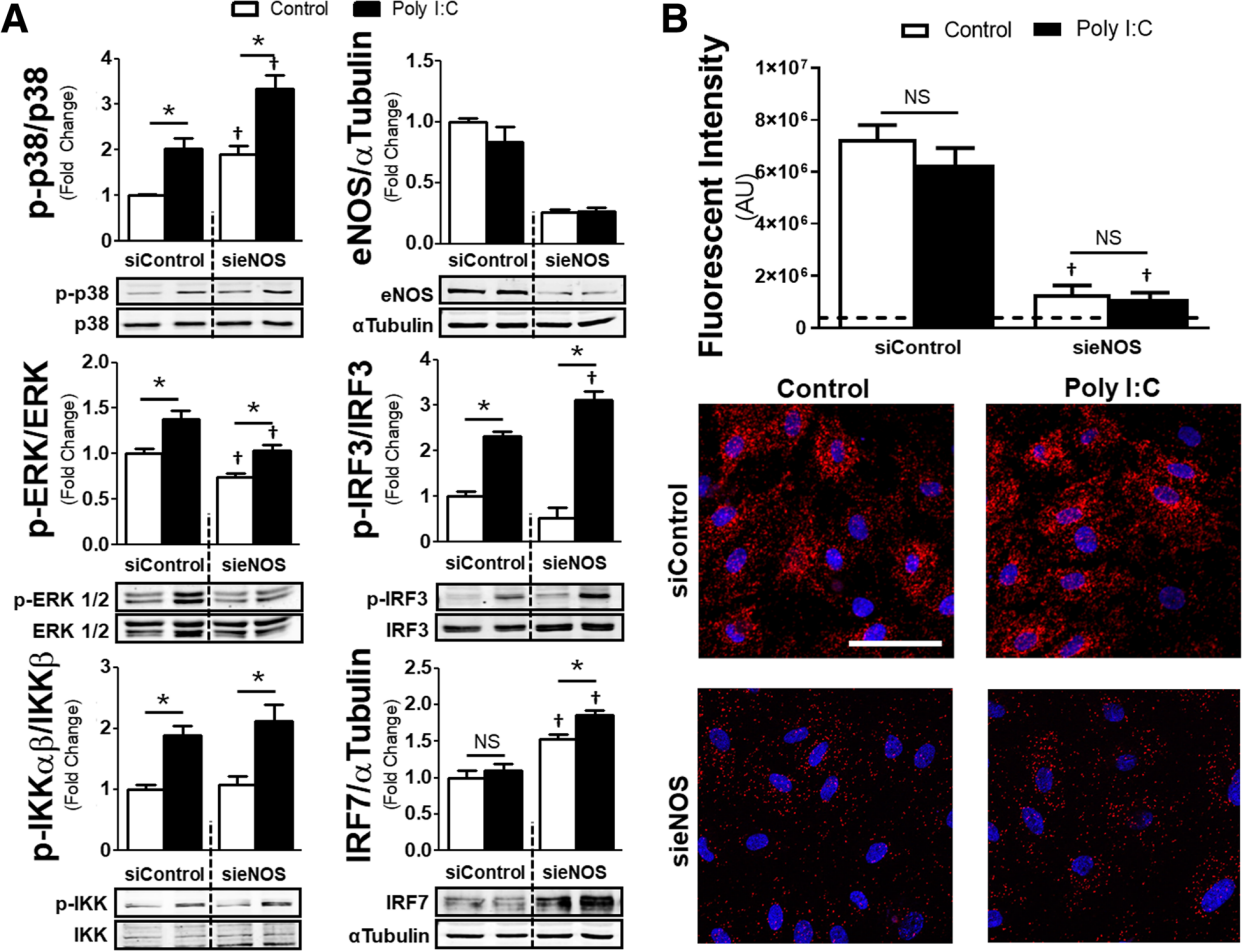
*Alterations in MAPK, IRF phosphorylation and p38-eNOS proximity ligation signal in eNOS knockdown*: **a** Normalized protein ratios and associated western blot images of HMVECs exposed to siRNA (siControl or sieNOS) for 72 h and then treated with Poly I:C (10 μg/mL) for 90 min. Images show band intensity for p-p38, p38, p-ERK 1/2, ERK 1/2, p-IRF3, IRF3, p-IRF7, IRF7, eNOS or α-tubulin with ratios of phosphorylated protein to respective total protein or eNOS to α-tubulin (n = 4 replicates per group). **b** Total fluorescent signal (intensity) using proximity ligation assays between cells exposed to siRNA (siControl or sieNOS) for 72 h and then treated with Poly I:C (10 μg/mL) for 90 min (*top*). Representative images of PLA signal (red) with nuclear stain (blue) for the different PLA treatment groups (*bottom*). Scale bar indicates 60 μm (*n* = 7 replicates per group). * = *p* < 0.05 between compared groups, † = p < 0.05 between group compared to respective siControl group, NS = non-significant

Potentiation of cellular inflammation in relation to eNOS knockdown has been previously described after TLR4 stimulation. To test whether similar intercellular signaling effects would occur after TLR3-mediated inflammation, we treated HMVECs with siRNA for 72 h and then challenged them with Poly I:C or vehicle control for 90 min prior to collecting cell lysates (Fig. 3a). Application of Poly I:C increased both phosphorylation of p38 and extracellular signal–regulated kinases (ERK) 1/2 in the siControl groups, two MAPKs known to interact with eNOS [15]. Further, Poly I:C treatment in the siControl group increased phosphorylation of IκB kinase (IKK), an important regulator of the canonical nuclear factor-κB (NF-κB) pathway, as well as interferon regulatory factor (IRF) 3, which is responsible for interferon-β production. However, IRF7, which is a late producer of interferon, was not altered. These data are in comparison to the sieNOS group, which also had increased phosphorylation of p38 after Poly I:C, though both the control and Poly I:C treatment groups had elevated levels of phospho-p38 compared to the siControl group. In contrast, the sieNOS group had reduced levels of phospho-ERK 1/2 compared to the siControl group and there was no significant changes between the sieNOS and siControl group with regards to TLR3-mediated IKK phosphorylation. Similar to p38, eNOS knockdown enhanced phospho-IRF3 levels and also enhanced total IRF7. We were unable to detect any changes in phospho-IRF7 after Poly I:C at the time point tested, which is consistent with previous data in endothelial cells [12].

In a separate set of experiments, we attempted to examine the temporal relationship of the observed inflammatory changes to the phosphorylation of eNOS or the induction of inducible NOS (iNOS). However, we were unable to detect any significant changes in phospho-eNOS at serine 1177, the phosphorylation site associated with eNOS-mediated NO production, nor were we able to detect the presence of any iNOS in the samples (Additional file 1: Figure S1 and S2). We were only able to detect a reduction in phospho-eNOS at 16 h after Poly I:C, which corresponded to a reduction in total eNOS at the same time point. The findings of a lack of iNOS induction and impaired eNOS phosphorylation are consistent with prior studies in microvascular endothelial cells undergoing infectious challenge [8, 16, 17]. To further correlate NO levels with the observed inflammatory potentiation, we utilized the fluorescent probe DAF-2 DA to measure intracellular NO production. While the application of L-NAME reduced measurable intracellular NO compared to control, the application of Poly I:C for 90 min or 6 h only induced a trend toward reduced intracellular NO at 6 h which was not statistically different compared to control conditions (Additional file 1: Figure S3).

To further explore if there was a direct relationship be p38 and eNOS, we utilized proximity ligation assays (PLA). To ensure that the signal was the result of protein-protein interactions between eNOS and p38, we exposed cells to siControl or sieNOS for 72 h. Afterwards, cells were exposed to Poly I:C for 90 min and PLA was performed (Fig. 3b). In both control conditions and after Poly I:C exposure, the assay demonstrated signal for eNOS-p38 protein-protein interactions, though those interactions did not differ between agonist groups. The exposure to siRNA to eNOS significantly reduced the PLA signal, suggesting the absence of off-target effects.

### p38 is a regulator of the endothelial response to TLR3

**Fig. 4:**
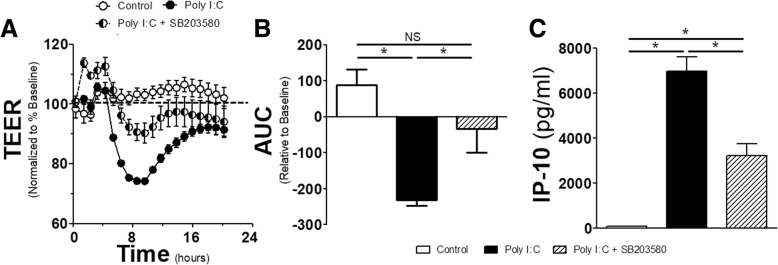
*Effect of the p38 inhibitor on endothelial resistance and IP-10 production*: **a** HMVECs were grown on transwells for 24 h then given SB203580 (10 μmol/L) or vehicle control (DMSO < 0.1%). 30 min later cells were exposed to Poly I:C (10 μg/mL) or control and TEER was recorded for every hour for 16 h afterwards and normalized to resistance 1 h prior to SB203580. **b** Associated AUC for duration of the TEER experiment in A. **c** IP-10 production as measured by ELISA for HMVECs exposure to combinations of SB203580 and Poly I:C at the time and concentrations previously stated (n = 4 replicates per group). * = *p* < 0.05 between compared groups, NS = non-significant

While several MAPKs exist in regulating inflammation in various cell types, p38 has been shown to be an important regulator of TLR responses in endothelial cells. Additionally, inhibition of p38 has been demonstrated to mitigate the effects of inflammation on endothelial permeability [18]. Given this, we sought to explore the effects of p38 on TLR3-mediated as well as eNOS-regulated permeability. 30 min prior to the exposure of Poly I:C, a p38-inhibitor (SB203580) or vehicle control (DMSO) was applied. The application of SB203580 increased the resistance of the endothelial monolayer and this increased resistance blunted the impact of Poly I:C through the remainder of the experiment (Fig. 4a). The total change in resistance relative to baseline, shown as AUC in Fig. 4b, demonstrated that Poly I:C induced a significant impairment in endothelial monolayer integrity, and that this impairment was mitigated by prior application of SB203580. To provide further relevance of the importance of p38 in TLR3-mediated inflammatory output, we measured IP-10 levels in the supernatants collected from cultured cells (Fig. 4c). While Poly I:C produced a large amount of IP-10 16 h after exposure, pre-treatment with SB203580 reduced the production by half.

### Pro-inflammatory changes by eNOS knockdown are mediated by p38

**Fig. 5:**
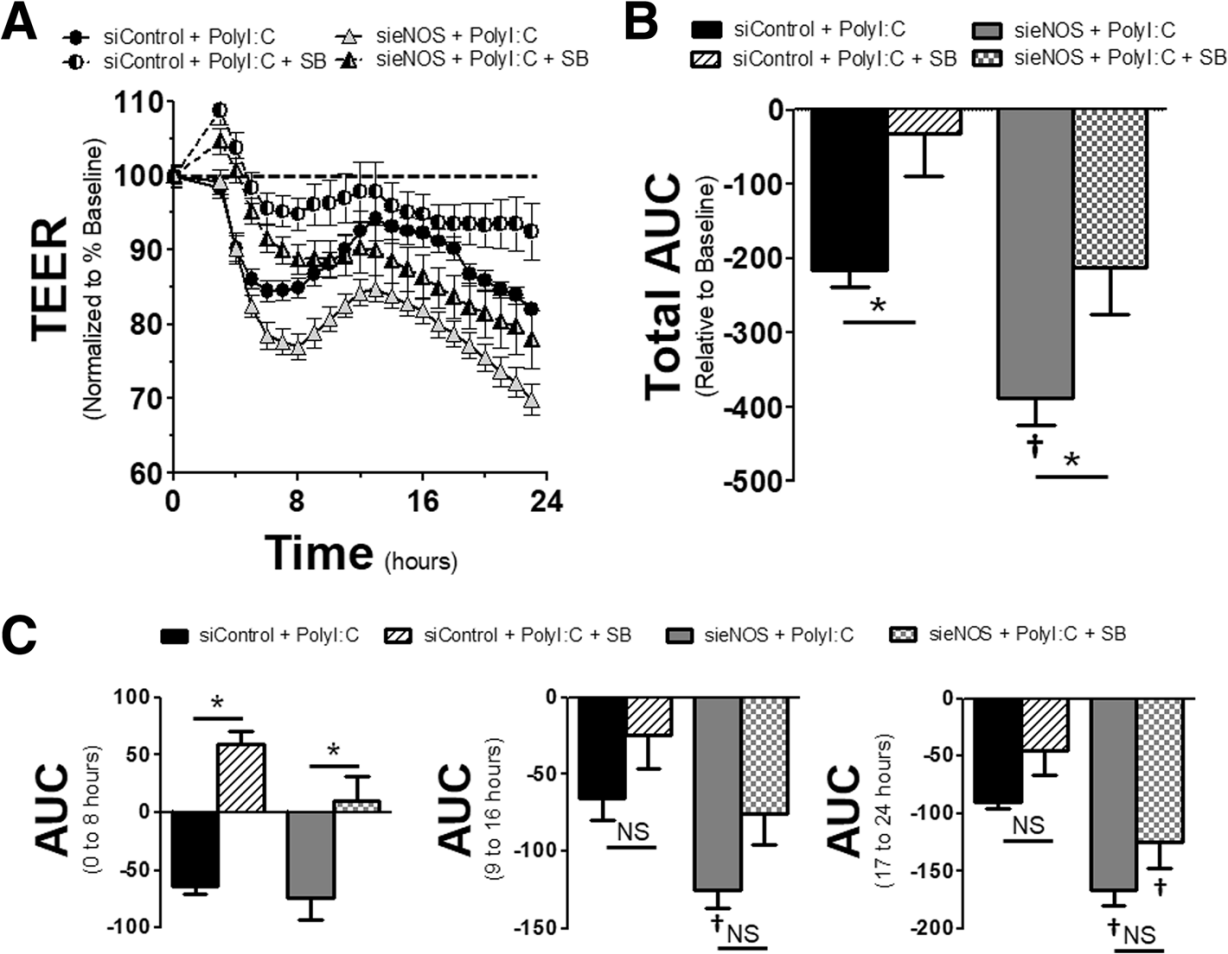
*eNOS knockdown and p38 inhibition alterations on endothelial resistance*: **a** HMVECs were exposed to siRNA for 72 h (siControl or sieNOS). 30 min prior to Poly I:C exposure (10 μg/mL), cells were treated with SB203580 (SB, 10 μmol/L) or vehicle control (DMSO < 0.1%). Trans-endothelial electrical resistance (TEER) was determined by measuring resistance across the monolayer at 1 h intervals for a total of 24 h across the respective groups and normalized to the resistance 1 h prior to agonist treatment. **b** Total area under the curve (AUC) for changes in resistance (increase = positive, decrease = negative) relative to baseline for all data points collected. **c** AUC for time intervals of 0 to 8 h (*left)*, 9 to 16 h (*center*) and 17 to 24 h (*right*) (*n* = 6 replicates per group). * = *p* < 0.05 between compared groups, † = *p* < 0.05 between group compared to respective siControl group, NS = non-significant

To examine the effects of p38 on eNOS knockdown, HMVECs were exposed to siRNA to eNOS or scrambled siRNA for 72 h. 30 min prior to Poly I:C exposure, cells were either treated with SB203580 or vehicle control. Application of SB203580 blunted the changes in TEER for both siControl and sieNOS groups after Poly I:C, relative to DMSO controls (Fig. 5a and b). Examining the dynamic effects of this, the most significant reduction occurred in the first 8 h after Poly I:C exposure (Fig. 5c). Here, the application of SB203580 significantly reduced the impact of Poly I:C on the AUC for both the sieNOS and siControl groups. In addition, there was no significant difference between sieNOS and siControl within their respective treatment groups, though there was variability in the replicates. In the 9 to 16 h time frame, the effect of Poly I:C on the AUC was reduced and no longer statistically significant, however, the sieNOS with vehicle treatment group had more change in the AUC compared to the siControl with vehicle treatment group. By the 17 to 24 h period, there continued to be no statistical significant effect of the SB203580 versus DMSO treatments, but those cells exposed to sieNOS had a significantly larger change in AUC compared to the siControl cells.

**Fig. 6:**
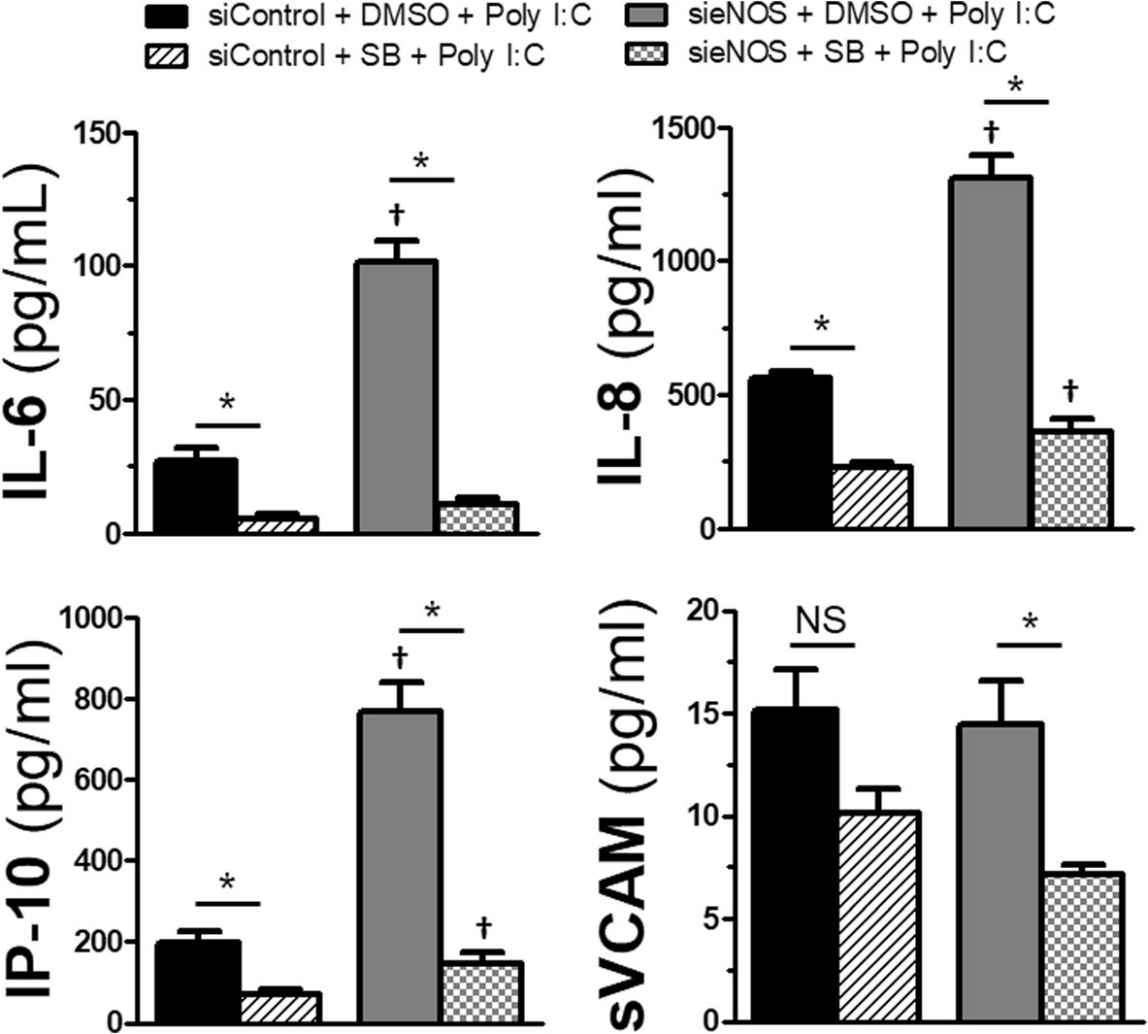
*Effect of eNOS knockdown and p38 inhibition on cytokines*: HMVECs were exposed to 72 h of siRNA (siControl or sieNOS) and then treated with SB203580 (SB, 10 μmol/L) or vehicle control (DMSO < 0.1%). After 30 min, cells were exposed to Poly I:C (10 μg/mL) for 6 h. Supernatants were collected and levels of IL-6, IP-10, IL-8 and sVCAM were determined by ELISA (*n* = 4 individual replicates). * = p < 0.05 between compared groups, † = p < 0.05 between group compared to respective siControl group, NS = non-significant

Next, we turned our attention to cytokine and chemokine production as well as release of soluble adhesion molecules, the latter which have been shown to correlate with viral infections and cardiovascular disease [19, 20]. Using the same duration of siRNA exposure of 72 h, and a 30 min pretreatment with SB203580 or vehicle control, we challenged cells with Poly I:C for 6 h before collecting supernatants. The shorter exposure time was done to correlate more with the timing of maximal permeability after TLR3 stimulation seen in the TEER experiments. Application of SB203580 prior to Poly I:C induced a significant reduction in the levels of IL-6, IL-8 and IP-10 compared to vehicle controls in the siControl group (Fig. 6). Likewise, in the sieNOS group exposed to SB203580, there was significant impairment of IL-6, IL-8 and IP-10 levels compared to vehicle controls. However, the cytokine and chemokine levels in the sieNOS with vehicle control group were 1 to 2-fold higher compared to the siControl group, again demonstrating that the loss of eNOS potentiated inflammation after TLR3 challenge. Though sVCAM production was slightly impaired by p38 inhibition, there was only a statistically significant effect in the sieNOS group and the sieNOS groups did not differ from the siControl groups as far as production.

### TLR3 and eNOS share a relationship in human biopsy specimens

**Fig. 7:**
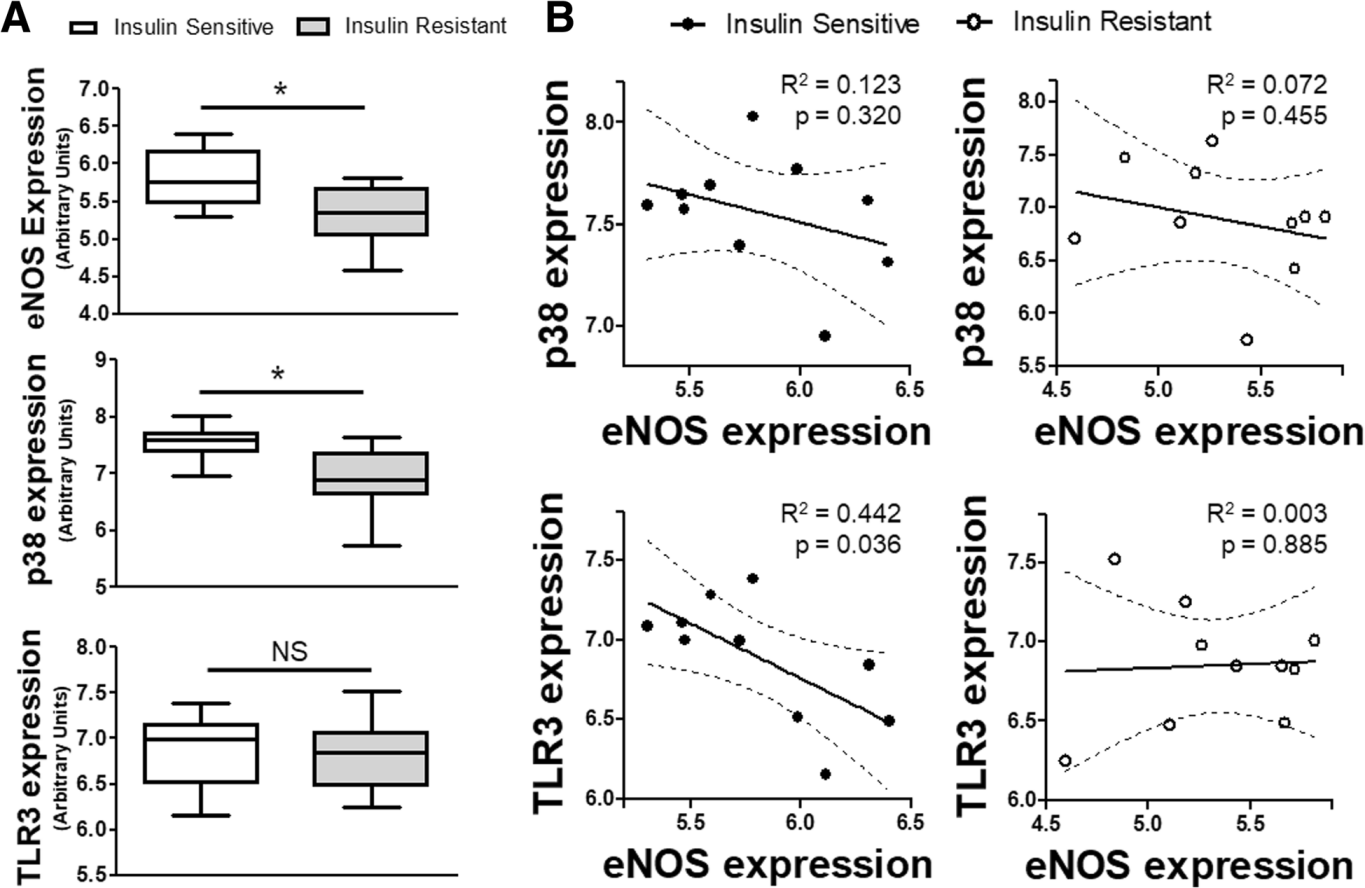
*eNOS, p38 and TLR3 expression in insulin sensitive and insulin resistant patients:* **a** Relative gene expression for eNOS, p38 and TLR3 from omental tissue biopsies obtained from insulin sensitive and insulin resistance patients (*n* = 10 per cohort). **b** Linear regression analysis (solid line) with 95% CI (dashed lines) for matched pair expression of p38 and eNOS or TLR3 and eNOS for insulin sensitive (*left*) and insulin resistance (*right*) cohorts. p and R^2^ values of regression analysis as shown. * = *p* < 0.05 between designated groups. NS = non-significant

Given our previous data examining the relationship of eNOS-TLR3-p38 in cultured human cells, we wanted to explore if any relationship existed in human patients. We queried the NCBI GEO database for insulin resistance, given the clinical association between insulin resistance and endothelial dysfunction, as well as the increased risk of infections in diabetic populations [21, 22]. We used dataset GSE20950, obtained by Hardy, et al. from omental adipose tissue collected from obese individuals who were sensitive to insulin and individuals who were resistant [13]. In the cohort of patients with insulin resistance, both the eNOS and p38 expression were decreased compared to the insulin sensitive cohort. The expression of TLR3 was no different (Fig. 7a). When matching individual patients to their own gene expression within their respective insulin sensitivity cohorts, we found that within the insulin sensitive group, higher eNOS expression was associated with lower TLR3 expression (Fig. 7b). This linear relationship was absent in insulin resistance patients who had lower baseline eNOS expression, suggesting the potential protective relationship was lost. With regard to p38, though both insulin sensitive and insulin resistant patients had a negative correlation between p38 and eNOS, neither subset achieved statistical significance by regression analysis.

## Discussion

Nitric oxide has played a controversial role in the development of endothelial inflammation, particularly in the setting of severe, acute infections [7, 23]. Much of this controversy stems from near opposing effects of NO depending on its source of production. This confusion has been enhanced by animal studies showing both detrimental and beneficial effects of NO production by NOS, with subsequent clinical studies showing increased mortality in septic patients given non-selective NOS inhibitors [24–27]. To further understand the role and mechanism of eNOS in endothelial cells during infectious challenge, we used several physiologic endpoints in human endothelial cells to determine what the effect of eNOS knockdown would be on TLR3-mediated inflammation by Poly I:C, a synthetic mimetic of dsRNA viruses. Application of siRNA to knockdown eNOS was associated with increased cytokine and chemokine production after Poly I:C exposure, but without differences in soluble adhesion factors. Knockdown of eNOS also produced a reduction in the baseline resistance of the endothelial monolayer that was exacerbated by Poly I:C. The effect of sieNOS induced significant alterations in MAPK signaling including a reduction in ERK 1/2, but potentiation of p38, IRF3 and IRF7. The importance of eNOS-p38 interactions was highlighted by the application of a pharmacologic inhibitor of p38 that reduced the effects of Poly I:C on resistance and cytokine secretion potentiated by eNOS knockdown. In addition, using PLA, eNOS and p38 were shown to have a direct interaction that was impaired by eNOS knockdown. Lastly, using visceral fat tissue from patient with diabetes, a disease known to impair eNOS and one that is associated with increased viral infection risk, we found correlations between p38, eNOS and TLR3 expression, suggesting an in vivo relationship. Together, these data suggest an important role of eNOS in regulating p38-mediated endothelial inflammation during viral infections.

It has been demonstrated in previous studies that the loss or reduction of eNOS can have negative consequences during infectious challenges, particularly after TLR4 activation [8, 28]. However, TLR4, the toll-like receptor for the bacterial cell wall component known as lipopolysaccharide, relies on a different intracellular signaling mechanism in endothelial cells than TLR3, the toll-like receptor responsible for inflammatory activation by dsRNA viruses [12, 29]. To test if the impact of eNOS was conserved across another TLR that utilizes a different intracellular signaling mechanism, we knocked down eNOS in human endothelial cells and challenged them with Poly I:C. The reduction of eNOS potentiated the TLR3-mediated release of cytokines and chemokines as well as exacerbated the reduction in monolayer resistance, a surrogate of permeability. This enhanced inflammatory response through the loss of eNOS was related to increased phosphorylation of p38 and IRF3 and increased production of IRF7. However, though TLR3 activation induced phosphorylation of IKK, the knockdown of eNOS did not potentiate this response. The reasons for this are unclear but maybe a product of the timing of sample collection, or the activation of alternative pathways such as the non-canonical NF-κB or activator protein-1 pathways [30, 31]. These data are congruent with previous data showing that eNOS knockdown potentiates the endothelial response to TLR4 stimulation for both cytokine production and intercellular adhesion [8]. Certainly, this protective role of eNOS in response to infectious challenge would offer an explanation as to why eNOS overexpressing animals are protected against endotoxic shock and why clinical trials of non-selective NOS inhibitors were detrimental [24, 32]. It is worth nothing that while L-NAME reduced detectable intracellular NO levels, it had no significant effect on TLR3-mediated inflammation as measured by cytokine production or permeability. Additionally, Poly I:C did not induce iNOS nor phosphorylation of eNOS. While certainly TLR3-mediated inflammation did not appear to be driven by enhanced NO production, it remains unclear how much the loss of basal NO production contributes to the pro-inflammatory phenotype. Our observations counter prior data that suggest the loss of eNOS can have a positive impact on vascular permeability after exposure to vascular endothelial growth factor and platelet activating factor [33, 34]. These dichotomous observations could be due to methodological differences or an effect of subtypes of endothelial cells that display regional phenotypes of permeability [35]. Alternatively, these differences may be related to direct changes in eNOS by VEGF or PAF, which are hypothesized to exert their NO-mediated effects through intracellular redistribution of eNOS, altering its effects [36]. In TLR-mediated signaling, the effects of activation on eNOS are less clear and may instead point toward a protective role that is more dependent on relative eNOS abundance which regulates global inflammation via direct protein-protein interactions and less dependent on NO production [8, 37, 38]. To further examine mechanisms of eNOS-mediated alterations in TLR3 inflammation, we focused on p38.

Regulation of endothelial-mediated vasculopathy is a complex process in which MAPKs, and particularly p38, play an integral role [39, 40]. In addition, it has been demonstrated that with regards to TLR-mediated inflammation, p38 serves an equally important role [8, 18, 41]. Given our data showing that up regulation of p38 phosphorylation was associated with a reduction in eNOS, we tested the role of p38 activity in mediating inflammation after eNOS knockdown. Application of a pharmacological inhibitor to p38, SB203580, abrogated a significant amount of the change in monolayer resistance after Poly I:C exposure. Further, p38 inhibition reduced the impact of eNOS knockdown on monolayer resistance either with or without TLR3 stimulation as well as reduced the pro-inflammatory cytokine and chemokine production in Poly I:C treated cells. To further give plausibility to an eNOS-p38 relationship in human disease, we examined gene expression in patients with obesity-related insulin resistance, which is linked to eNOS-mediated vascular dysfunction. Indeed, altered MAPK and TLR signaling with reduction of eNOS are hallmarks of chronic inflammation seen in diet-related diabetes [37, 42]. In patients with insulin resistance, eNOS, as well as p38, gene expression were reduced. In addition, there existed a linear relationship between eNOS and TLR3 expression, with higher TLR3 expression in those individuals with less eNOS expression. Though gene expression does not yield the same information as protein levels nor protein activity and can have mixed interpretation in relation to acquired diseases, the association gives credence to the hypothesis that reduced eNOS expression serves an important role in mediating vascular inflammation, including that seen in chronic and acute inflammation.

A lack of significant NO alterations coupled with the exacerbated p38 phosphorylation led us to explore if direct eNOS-p38 interactions could be a plausible hypothesis for the observed inflammatory phenotype seen after eNOS knockdown. Specific to this direct relationship, an association was initially demonstrated in isolated eNOS and MAPK proteins [15]. In that study, both fixed p38 and ERK were shown to physically interact with eNOS in solution and the kinetics of disassociation happened both naturally at a slow rate, and in a calcium-dependent manner at a faster rate. It is worth noting that these binding associations were either absent or reduced with neuronal NOS, another constitutively expressed NOS isoform, suggesting unique properties of eNOS-MAPK interactions. These interactions were postulated to be due to the presence of a pentabasic MAPK docking site on eNOS (sequence Arg-Arg-Lys-Arg-Lys) which could bind to a conserved, reciprocal peptide sequence on the MAPKs referred to as the common docking domain [43]. It was later established that these associations of eNOS with p38 occur within living human endothelial cells as determined by immunoprecipitation [8]. We have further validated these findings to show direct proximity of eNOS and p38 within endothelial cells utilizing PLA, where eNOS-p38 associations were reduced after eNOS knockdown. These findings raise questions about the direct modulatory relationship between eNOS and MAPK and how these interactions regulate or are responsible for inflammatory vasculopathy, especially in the setting of infectious challenge for individuals who already carry a risk of impaired eNOS due to prior vascular disease.

## Conclusions

The data presented provide further evidence for the important role of eNOS in the regulation of MAPK-mediated endothelial inflammation. Further, the MAPK, p38, provides an integral role in the endothelial response to TLR3 challenge, the primary receptor for dsRNA viruses. The loss of eNOS under these conditions worsened the p38-mediated impact on cytokine production, such as IL-6 and IP-10, as well as impaired endothelial barrier integrity. This impact appeared to be partially dependent on a loss of direct interactions between eNOS and p38, suggesting an important immunomodulatory role of eNOS in both TLR3 and p38 signaling that may exacerbate the infectious response in individuals who are already at risk for vascular disease via altered eNOS levels. These data provide new insights into eNOS and MAPK interactions in the regulation in acquired vascular dysfunction.

## Additional file

Additional file 1: **Figure S1.** Lack of iNOS induction in human endothelial cells: HMVECs (*left*) were exposed to Poly I:C (10 μg/mL) for 90 minutes, 6 hours or 16 hours, lysates were collected and examined for iNOS expression by western blot. No bands were detectable at the estimated molecular weight of ~135 kD. For a positive control for the antibody, murine vascular smooth muscle cells (*right*) were exposed to TNFα (10 ng/mL) and examined for iNOS induction via western blot. Representative images are shown for a single experiment (n=4 replicates per agonist group). **Figure S2.** Temporal change in phospho-eNOS and total eNOS after Poly I:C: HMVECs were exposed to Poly I:C (10 μg/mL) for 90 minutes, 6 hours or 16 hours, lysates were collected and examined via western blot for phospho-eNOS at residue serine 1177 compared to total eNOS (*left*) or total eNOS compared to α-tubulin (*right*) at the respective time points. Normalized protein ratios are shown above representative images of a single comparison (n=4 replicates per time point). * = *p* < 0.05 between compared groups, † = *p* <0.05 between compared control, NS = non-significant. **Figure S3.** Amount of intracellular NO after L-NAME and Poly I:C treatments: HMVECs were exposed to L-NAME (100 μmol/L) or vehicle control (DMSO <0.1%) for 90 minutes and compared to HMVECs treated with Poly I:C (10 μg/mL) for 90 minutes or 6 hours. Cells were examined for intracellular NO production using the fluoroprobe DAF-2 DA (5 μmol/L) and total fluorescent intensity was normalized to control, untreated conditions. Scale bar indicates 60 micrometers (*n*=5 individual replicates per group). * = *p* < 0.05 between compared groups, NS = non-significant. (DOCX 106 kb)
