# Supplementary material for: Toll-like receptor 3-mediated inflammation by p38 is enhanced by endothelial nitric oxide synthase knockdown
Source: Cell Commun Signal. 2019 Apr 15;17:33. doi: 10.1186/s12964-019-0345-3 (PMC6466662; doi:10.1186/s12964-019-0345-3)
Supplement: Supplementary file 1 — Figure S1. Lack of iNOS induction in human endothelial cells: HMVECs (left) were exposed to Poly I:C (10 μg/mL) for 90 minutes, 6 hours or 16 hours, lysates were collected and examined for iNOS expression by western blot. No bands were detectable at the estimated molecular weight of ~135 kD. For a positive control for the antibody, murine vascular smooth muscle cells (right) were exposed to TNFα (10 ng/mL) and examined for iNOS induction via western blot. Representative images are shown for a single experiment (n=4 replicates per agonist group). Figure S2. Temporal change in phospho-eNOS and total eNOS after Poly I:C: HMVECs were exposed to Poly I:C (10 μg/mL) for 90 minutes, 6 hours or 16 hours, lysates were collected and examined via western blot for phospho-eNOS at residue serine 1177 compared to total eNOS (left) or total eNOS compared to α-tubulin (right) at the respective time points. Normalized protein ratios are shown above representative images of a single comparison (n=4 replicates per time point). * = p < 0.05 between compared groups, † = p <0.05 between compared control, NS = non-significant. Figure S3. Amount of intracellular NO after L-NAME and Poly I:C treatments: HMVECs were exposed to L-NAME (100 μmol/L) or vehicle control (DMSO <0.1%) for 90 minutes and compared to HMVECs treated with Poly I:C (10 μg/mL) for 90 minutes or 6 hours. Cells were examined for intracellular NO production using the fluoroprobe DAF-2 DA (5 μmol/L) and total fluorescent intensity was normalized to control, untreated conditions. Scale bar indicates 60 micrometers (n=5 individual replicates per group). * = p < 0.05 between compared groups, NS = non-significant. (DOCX 106 kb) [file 12964_2019_345_MOESM1_ESM.docx]

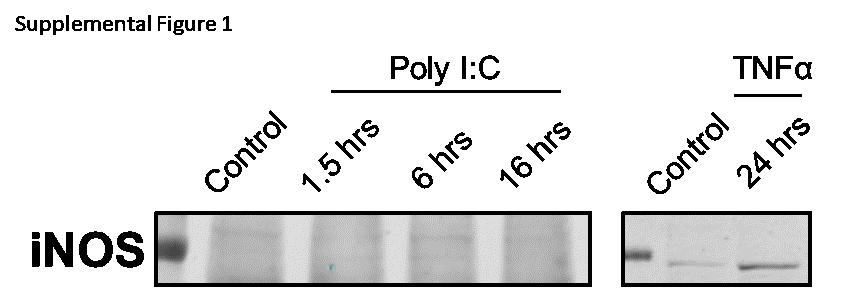


Supplemental Figure 1: *Lack of iNOS induction in human endothelial cells*: HMVECs (*left*) were exposed to Poly I:C (10 µg/mL) for 90 minutes, 6 hours or 16 hours, lysates were collected and examined for iNOS expression by western blot. No bands were detectable at the estimated molecular weight of ~135 kD. For a positive control for the antibody, murine vascular smooth muscle cells (*right*) were exposed to TNFα (10 ng/mL) and examined for iNOS induction via western blot. Representative images are shown a single experiment (n=4 replicates per agonist group).


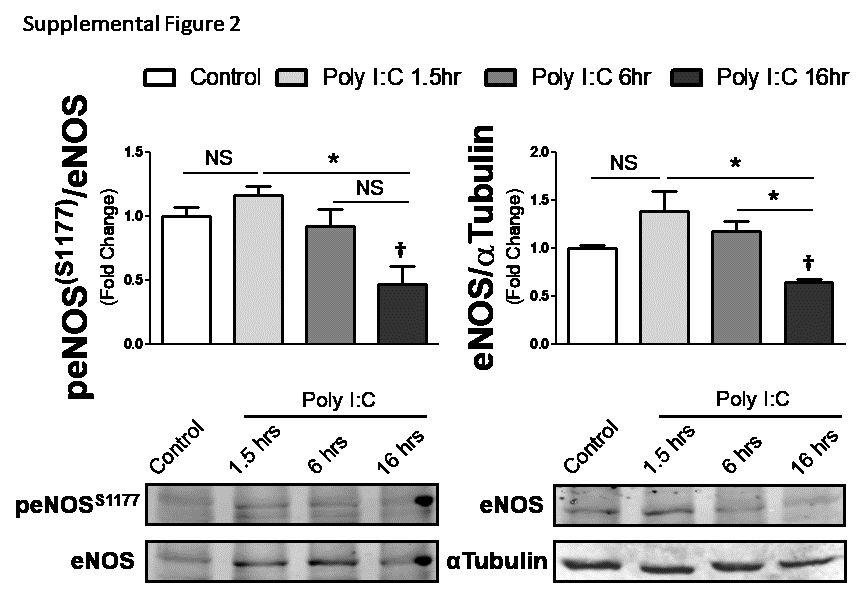


Supplemental Figure 2: *Temporal change in phospho-eNOS and total eNOS after Poly I:C*: HMVECs were exposed to Poly I:C (10 µg/mL) for 90 minutes, 6 hours or 16 hours, lysates were collected and examined via western blot for phospho-eNOS at residue serine 1177 compared to total eNOS (*left*) or total eNOS compared to α-tubulin (*right*) at the respective time points. Normalized protein ratios are shown above representative images of a single comparison (n=4 replicates per time point). * = p < 0.05 between compared groups, † = p <0.05 between compared control, NS = non-significant.


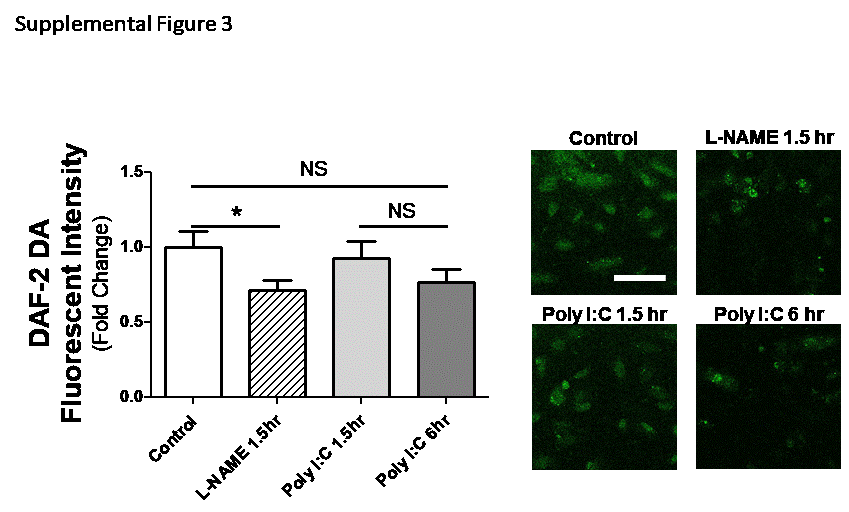


Supplemental Figure 3: *Amount of intracellular NO after L-NAME and Poly I:C treatments*: HMVECs were exposed to L-NAME (100 μmol) or vehicle control (DMSO <0.1%) for 90 minutes and compared to HMVECs treated with Poly I:C (10 µg/mL) for 90 minutes or 6 hours. Cells were examined for intracellular NO production using the fluoroprobe DAF-2 DA (5 μmol) and total fluorescent intensity was normalized to control, untreated conditions. Scale bar indicates 60 micrometers (n=5 individual replicates per group). * = p < 0.05 between compared groups, NS = non-significant.
